# Cases of, and Recent Operations upon, Necrosed and Carious Bones

**Published:** 1861-12

**Authors:** J. F. Miner


ART. IV.—*Cases of, and recent Operations upon, Necrosed and Carious Bones.*—By J. F. MINER, M. D.

My attention having been recently called to several cases of diseased bone, of long standing, which have been very successfully removed by operation, I have thought a brief history of some of them, and the results of treatment, might interest sufficiently, to be worthy of publication: this I am induced to do from the extreme frequency of necrosis, and caries, and the prevailing reluctance in surgeons to attempt its removal.

The severity of the operation, and the uncertainty which generally prevails as to the exact condition and character of the disease, and the tedious, and slow recoveries, which must generally follow, have no doubt induced many surgeons to advise delay, rather than the adoption of operative measures for relief; yet perhaps in no department of surgery is greater satisfaction afforded, or more pleasant results obtained, than in carefully considered, well performed operations for removal of diseased bone.

Capt. Stephen Champlin of Buffalo, aged 72, was wounded near Mackinaw, Sept. 3d, 1814, by a canister shot, a 1½ ounce iron ball passing through the thigh bone, near the middle, and remaining in the muscles eighteen days, during the three first of which, he was exposed in an open boat, without surgical aid. In November following after the ball was extracted, he was placed in a vessel and sent to Erie, Penn. In about five months he was so far recovered as to be able to travel by stage to Norwich, Conn. During the summer was examined by many eminent surgeons, among others Dr. Mott of New York, and was advised by all, to postpone operation, as but little pain was suffered and the discharge was but small in amount. In March, 1816, a small piece of dead bone was extracted which afforded some relief, and he was able to command a U. S. vessel employed in the survey of the boundary line between this country and Great Britain, on the Lakes. March, 1817, another piece of necrosed bone was extracted, which also gave some temporary relief from the pain and discharge. In 1824

Prof. Nathan Smith of Yale College, made an operation, removing quite a large piece of bone which was in process of exfoliation; it was regarded by him as probably the removal of the disease, and though unsatisfactory in this respect, it was attended by great relief. The year following another piece of bone was removed, and in 1859 a small piece was discharged from the same place.

Upon examination the leg is shortened about three inches; knee joint anchylosed; great enlargement of the lower portion of femur, and upon the outer side of the leg a festulous opening which communicates with the bone near the junction of the lower, with the middle third, the probe seeming to pass deeply into the bone. Pain at times very severe; constant discharge of thin unhealthy pus, in small quantity. Nov. 14, 1861, sulphuric ether being administered, assisted by Drs. Rochester, Lothrop Mason, King, and medical students, incision was made to the bone, exposing it freely; following the apparent direction of the small opening with a trephine we passed to the interior, through the newly formed healthy bone, a distance of near an inch, finding through this secure and remarkably dense and thick covering a piece of necrosed bone, the cause of this protracted disease and pain. Nature had accomplished very much in throwing off the exfoliated portions which had from time to time been removed, but unassisted could never free itself of this "leven of misery." The small vessels bled very freely, and we were obliged to apply ligature to a good many; in other respects the operation was unattended by serious difficulty. The patient soon rallied from the effects of ether; the loss of blood did not greatly depress, and he seems thus far to be comfortably and thoroughly relieved, of the principal cause of pain, which he has suffered more or less severely, for forty-seven years.

J. Pernett, aged 20, robust and naturally healthy, received some slight injury, or strain, some ten years since, to which he attributes his present condition and past troubles. Soon after this injury his left thigh commenced to swell, and after a short time an abscess formed and discharged pus, which has never ceased for any length of time since; he is somewhat reduced in strength by the pain and constant discharge, but otherwise is in excellent health, with no known hereditary predisposition to disease. After careful examination aided by Prof. J. R. Lothrop, it was proposed to make exploratory operation, and if practicable remove whatever of dead bone might be found; this plan was adopted, since the disease unless removed by operation must certainly be very lasting and distressing, possibly even fatal, by inducing other forms of disease.

Nov. 2d, 1861, assisted by Professors Lothrop and Mason and medical students; chloroform being administered, a probe was passed through the external opening down to and finally into the femur, upon the inner side, just above the inner condyle. A free opening was now made, and finding the sinus in the bone, it was enlarged with a triphine, and an opening thus made directly to a great quantity of diseased and decayed bone, which was easily and thoroughly removed.

Nov. 20. This patient has nearly recovered from the operation in every respect. The time has not yet been sufficient to allow the bone to take on healthy granulation and heal over, consequently there is yet some slight discharge, but in other respects the patient is well. A small tent has been kept in the wound to prevent external healing, until the wound might granulate from the bed of disease in the bone, which it appears to be rapidly doing, giving every promise that in a few weeks the patient will be wholly free from disease.

William Russell, aged 65, formerly of good constitution, injured by a fall in 1840, fracturing his leg; of the exact nature of the fracture am not informed; had been able to labor up to 1855, but has never been free from pain and discharge since the injury.

Leg is greatly swolen; tibia exposed for a distance of three inches, and carious; probe passes a short distance into the softened bone. Pain, discharge, and other depressing influences, have greatly reduced the health and strength insomuch that operation for relief is very hesitatingly decided upon.

Feb'y 9, 1861. Chloroform being administered, the tibia was freely exposed, and with great care all the carious portion was removed, requiring the larger part of the upper portion to be cut away, leaving an irregular but healthy surface. Healthy granulations appeared from the bone surface, and in about eight months from the time of operation he had so fully regained his health and strength as to be able to pursue his usual occupation.

---

*Subnitrate of Bismuth in Gonorrhœa.*—Dr. Moulson confirms, in the *Recueil de Mem. de Med. Milit.*, the good account given by Caby of the efficacy of this treatment. Me mixes twenty parts of well washed bismuth in two hundred parts of water, throws some of this into the urethra and has it there retained for ten minutes, a local emollient bath having been employed previously. In only the severest cases is the patient obliged to maintain absolute rest for four or five days, after which he is enabled to return to his ordinary habits and diet. A cure is said to take place more quickly than under the use of any other agent.—*Med. Times and Gazette.*

